# The effect of working memory load on selective attention to emotional faces for social anxiety individuals

**DOI:** 10.1002/pchj.736

**Published:** 2024-02-01

**Authors:** Mingfan Liu, Chen Cheng, Yating Xu, Lirong Zeng

**Affiliations:** ^1^ Department of Psychology Jiangxi Normal University Nanchang China; ^2^ Center of Mental Health Education and Research Jiangxi Normal University Nanchang China

**Keywords:** emotional face, flanker task, selective attention, social anxiety, working memory load

## Abstract

Research has confirmed that individuals with social anxiety (SA) show an attentional bias towards threat‐related stimuli. However, the extent to which this attentional bias depends on top‐down cognitive control processes remains controversial. The present study investigated the effect of working memory (WM) load on selective attention to emotional faces in both high social anxiety (HSA) and low social anxiety (LSA) groups by manipulating WM load through the inclusion of forward counting in multiples of two (low load) or backward counting in multiples of seven (high load) within a modified flanker task. In the flanker task, emotional faces (angry, happy, or neutral faces) were used as targets and distractors. A total of 70 participants (34 HSA participants; 36 LSA participants) completed the flanker task in the laboratory. The results showed that the HSA individuals performed worse when responding to angry targets. Relative to LSA individuals, HSA individuals showed interference from angry distractors in the flanker task, resulting in significantly lower accuracy in identifying angry targets compared to happy targets. These results were unaffected by the manipulation of WM load. The findings imply HSA individuals have impaired attentional control, and that their threat‐related attentional bias relies more on the bottom‐up automatic attentional process.

## INTRODUCTION

Selective attention filters information, enabling people to focus on important stimuli relevant to the target. In general, selective attention processes involve two models of resource allocation. The bottom‐up attention is the stimulus‐driven and can be selected based on the salience of stimulus features relative to its surroundings (Woodman et al., 2013). However, the top‐down attention is known as the goal‐directed system that allows us to focus limited‐capacity processing on task‐relevant information despite the lack of saliency in stimulus features (Wolfe, 1994). Cognitive control can provide a good explanation for goal‐directed attention regulation mechanism. It can flexibly adjust the distribution of attention according to the current target stimuli, so that individuals can maintain target attention and inhibit habitual reactions or impulsive behaviors in the presence of distractors (Giovanni, 2014; Muris et al., 2008).

Emotional information plays a relatively important role in determining the allocation of attentional resources, thus influencing the process of learning and memory (Seli et al., 2016; Zinchenko et al., 2019). For example, emotional faces, which hold significant adaptive value in conveying essential social information and potential benefits or dangers, have been demonstrated to influence the allocation of selective attention (Chen et al., 2016; Lazarov et al., 2016; Ran & Chen, 2017; Vuilleumier & Schwartz, 2001).

Anxiety about being negatively judged in social interaction is the major symptom of social anxiety disorder (Heimberg et al., 2014). Considering the sensitivity of individuals with social anxiety (SA) to negative social evaluation, threatening facial expressions have special clinical significance for them (Rapee & Heimberg, 1997). Angry faces are the most prominent example of threatening stimuli because angry expression is regarded as the most obvious sign of criticism and hostility in social situations (Ekman, 1973). Some studies have shown that SA individuals are particularly sensitive to angry faces (Liang et al., 2017; Mogg et al., 2004; Moriya & Tanno, 2011). For example, within a specific time interval of 250–1000 ms when SA individuals were presented with pictures of angry, happy, neutral, and sad faces at the same time, they had higher fixation probability and longer maintenance time for angry faces (Liang et al., 2017). Typically, this threat‐related attentional bias is considered to primarily rely on bottom‐up attentional processes. Consistent with this view, the evolutionary models hold that bottom‐up processing of threatening social cues is functional and automatic (Fox et al., 2002; LoBue et al., 2010).

Interestingly, research has found that the threat‐related attentional bias does not always rely on automatic bottom‐up attention, and may also require the participation of top‐down attention. In a visual search task, participants were asked to detect the target faces as quickly as possible while ignoring distractors. The results found that the search times for negative faces increased with the number of distractors (Eastwood et al., 2001). Because the process of automatic detection in visual search is considered independent of changes in the number of distractors (Treisman & Gelade, 1980), it is reasonable to speculate that the recognition of threatening stimuli for humans is not completely resource‐independent, and that this process may have a more complex top‐down mechanism. However, the impairment of cognitive control functions plays a very important role in the etiology and symptom maintenance of SA (Morrison & Heimberg, 2013). Thus, it remains to be further explored whether attention to threat‐related stimuli in SA individuals depends on top‐down cognitive control.

To date, manipulating working memory (WM) load during selective attention tasks has been a common research method to examine the role of cognitive control mechanisms in preventing distractor interference. High load reduces the availability of WM to maintain stimulus priority in selective attention tasks (de Fockert et al., 2001). Although many studies have explored how the WM load affects selective attention in SA individuals, they have yielded inconsistent results. For example, some studies have shown that increased WM load would lead to decreased attention control and greater difficulty for SA individuals or trait anxiety in escaping threat‐related distractors (Berggren et al., 2012; McKendrick et al., 2018). Interestingly, another study found that high (compared to low) SA individuals were more likely to be distracted by task‐irrelevant face stimuli (e.g., angry faces) and required longer reaction times to recognize target letters in visual search tasks under high cognitive load. However, they showed better accuracy than low SA individuals (Soares et al., 2015).

The inconsistent results in previous studies may be due to the fact that the WM load was not adequately manipulated. For example, McKendrick et al. (2018) informed participants about the speech task information (e.g., the participants were told that they would have to give a speech to the audience) before completing the anti‐saccade task to increase social‐cognitive load. However, the speech task increases the SA rather than the demands on WM, which is usually regarded as the process of maintaining and manipulating information in a short time (Baddeley, 1992). Furthermore, the visual search task used in most studies assesses attentional bias rather than attentional control (Soares et al., 2015). A more important way to explore cognitive control is to use tasks involving “conflict handling,” such as the flanker task (Kim et al., 2017). Although a recent study also used the modified flanker task to investigate the selective attention of SA individuals (Chen et al., 2016), this study did not control for participants' depression levels and did not consider WM load as a variable. These suggest that the top‐down cognitive control mechanisms associated with attentional bias in SA individuals remain unclear.

In summary, in order to investigate whether the attentional bias of SA individuals towards threat stimuli depends on top‐down cognitive control, the present study examined the effect of WM load on selective attention to emotional faces in SA individuals. Consistent with previous studies, the present study used a flanker task involving “cognitive conflict processing” (Eriksen & Eriksen, 1974; Kim et al., 2017; Zhou & Liu, 2013). The target stimuli always appeared in the center of the flanker task, while the distractors appeared to the left or right of the targets (Petrucci & Pecchinenda, 2016). Because the target and distractor stimuli appear simultaneously on each trial, the flanker paradigm creates a situation in which the flanker and target stimuli compete for attention, allowing attentional biases towards different types of flanker stimuli to be analyzed and compared (Tannert & Rothermund, 2020). The compatibility between targets (angry/happy faces) and distractors (angry/happy/neutral faces) was manipulated based on emotional valence. WM load was controlled using a novel continuous counting method in which participants were asked to simultaneously count forward in multiples of two (low load) or backward in multiples of seven (high load) during the flanker task (Petrucci & Pecchinenda, 2016). This method requires participants to continuously calculate, update, and monitor the information already in their minds, which is a significant challenge to cognitive control functions.

According to the attention control theory (ACT; Eysenck et al., 2007), an increase in WM load impairs attention control due to resource competition. Therefore, we predicted that participants would show reduced attention control ability (i.e., longer reaction times and lower accuracy in the flanker task) under high WM load compared to low WM load. Furthermore, if the attentional bias of SA individuals towards threatening stimuli is driven by bottom‐up attention, then this bias would be unaffected by the WM load manipulation in high social anxiety (HSA) individuals compared to low social anxiety (LSA) individuals. In contrast, if this bias of SA individuals is driven by top‐down attention, then this bias of HSA individuals would be attenuated under high WM load.

## METHODS

### Participants

All participants were recruited via campus notice boards and online questionnaires. All participants completed the Liebowitz Social Anxiety Scale (LSAS; Liebowitz, 1987), which was used to assess SA symptoms and confirm appropriate membership for each participant. Participants with LSAS score ≥ 56 were included in the HSA group, while those with LSAS score ≤ 26 were included in the LSA group (Chen et al., 2016). This diagnosis method was confirmed by structured clinical interviews for the Mini‐International Neuropsychiatric Interview (MINI; Sheehan et al., 1998) and the 11th edition of the *International Classification of Diseases* (ICD‐11; Khoury et al., 2017), which were administered by trained and experienced interviewers. The exclusion criteria for both groups were as follows: (1) suicidal intent or thoughts; (2) current drug abuse or dependence; (3) current or past mental illness, such as schizophrenia, mania, and depression; (4) and neurological diseases or other mental disorders. Additionally, all participants had not taken any psychiatric medication within the past 90 days. Subsequently, to control for possible confounding by depression, all participants were required to complete the Beck Depression Inventory‐II (BDI‐II; Beck et al., 1985) on the day of the experiment, and participants with a BDI‐II score greater than 20 were excluded. The mean score of the BDI‐II was 7.56 (*SD* = 3.85) for the HSA group, and 5.06 (*SD* = 3.21) for the LSA group.

Ultimately, the sample consisted of 35 HSA individuals and 37 LSA participants. The HSA group (LSAS mean = 79.62, *SD* = 15.36) and LSA group (LSAS mean = 17.56, *SD* = 3.85) differed significantly on the LSAS scale (*t*_(70)_ = 16.30, *p* < .001, Cohen's *d* = 4.36). However, one participant from each group was excluded due to accuracy below 50% on the counting task. Therefore, 34 HSA participants (16 males, 18 females, mean age = 19.76 years, *SD* = 1.92 years) and 36 LSA participants (17 males, 19 females, mean age = 19.83 years, *SD* = 2.18 years) were included in the final study. An a priori power calculation in G*Power (V. 3.1.9.2; Faul et al., 2007) indicated that 58 participants were required to detect a medium effect size (Cohen's *d* = 0.60) with a power of 0.8 at *α* = .05. All participants were right‐handed and had normal or corrected‐to‐normal vision. All participants signed the informed consent and received payment after study completion. The study was approved by the Ethics Committee of the Jiangxi Normal University.

### Materials

The greyscale images of 30 different male individuals and 30 different female individuals (mean age = 24 years, *SD* = 2.56 years, age range 18 to 33 years) were selected from the system of Chinese facial expressions of emotion (Liu et al., 2012). Each individual bore an angry, happy, and neutral expression (180 face stimuli in total). Each image was corrected using software such as Adobe Photoshop 6.0, FunMorph, and so forth, to remove background objects and other distracting elements in the image, leaving only the core faces, and was uniformly adjusted to black and white images of the same brightness to reduce the interference for the participants. Additionally, overt obvious mouth opening, strabismus, squinting and other details that might attract attention were corrected in the original image to reduce the impact of visual salience on the allocation of attention resources (Calvo & Lundqvist, 2008). Finally, to maintain the ecological validity of the image, the hair was kept low and the hair on the forehead did not cover the eyebrows and eyes.

To further verify the effectiveness of emotional faces processing, facial images which were rated on 9‐point Likert‐type scales for valence (ranging from 1 = *very negative* to 9 = *very positive*) and arousal (ranging from 1 = *very calm* to 9 = *very excited*), were completed by 42 participants (20 males, 22 females; mean age = 22 years, *SD* = 2.3 years, age range 19–27 years) during the image‐selection phase. Finally, the 132 emotional faces were selected to represent three valence categories: 44 angry faces (mean valance [*SD*] = 2.83 [0.50]; mean arousal [*SD*] = 5.41 [0.50]), 44 happy faces (mean valance [*SD*] = 6.49 [0.36]; mean arousal [*SD*] = 5.40 [0.66]), and 44 neutral faces (mean valance [*SD*] = 4.35 [0.34]; mean arousal [*SD*] = 4.74 [0.22]). The three types of emotional faces differed significantly from each other in valence (*p* < .001). However, for arousal, happy faces and angry faces were significantly more arousing than neutral faces (*p* < .001), and happy faces and angry faces did not differ from one another in arousal (*p* > .05). Twelve emotional faces were used in the practice session, while 120 emotional faces were used in the experimental session.

### Procedure

Participants were seated in front of a computer screen at a distance of approximately 60 cm in a quiet laboratory. Pictorial stimuli (resolution 1920 × 1080 pixels, refresh rate 60 Hz) were presented on a black background using E‐Prime 2.0, maintaining a fixed height of 3.4 cm (3.26°× 3.26°of visual angle).

Participants were asked to perform two tasks simultaneously: the emotional flanker task and the counting task. The counting task was designed to create different levels of cognitive load and consisted of two different tasks. When a two‐digit number was displayed on the screen, the participants had to count forward in steps of 2 (low WM load). Conversely, when a three‐digit number was displayed on the screen, participants had to count backwards in steps of 7 (high WM load).

Participants were required to perform two tasks in parallel and to prioritize throughout the study. Participants first completed 30 practice trials before entering the formal experimental session. The formal experiment consisted of 10 blocks of 60 trials each. The trials reflected the combination of the following conditions: target face (2: angry, happy), distractor face (3: angry, happy, neutral), and WM load (2: low, high). For each block, the position of the target and distractor faces was equally probabilistic. There was a 2‐min rest interval between each of the two blocks, and experimental trials were presented in a randomized order.

**FIGURE 1:**
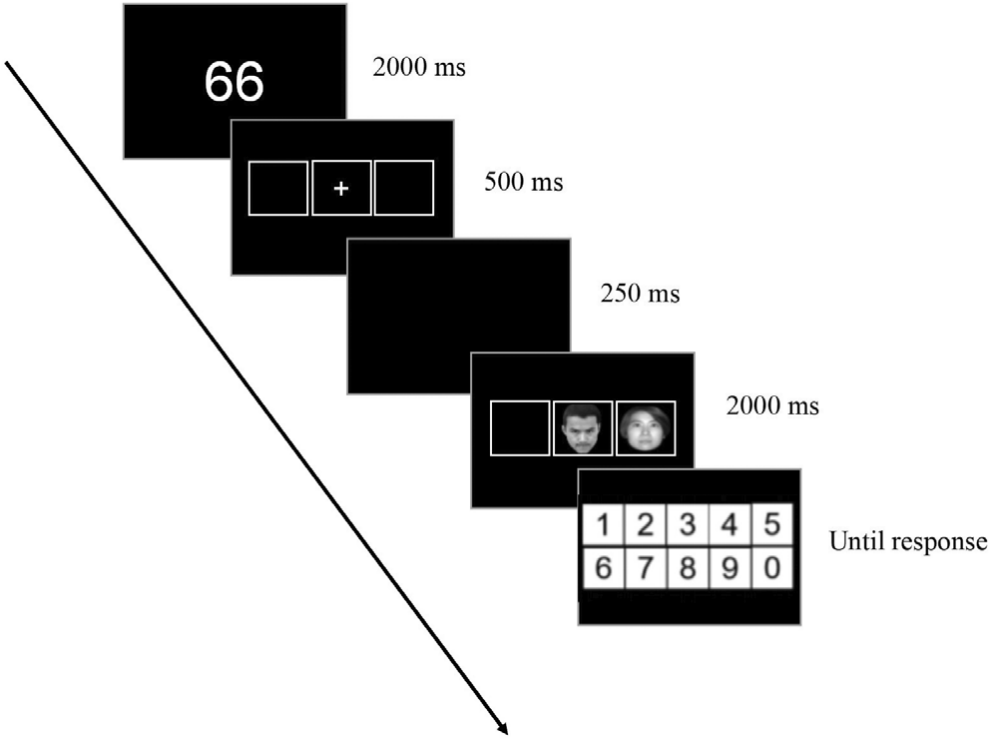
A typical trial sequence in the study: The example shows an angry target face flanked by a neutral distractor face under low working memory (WM) load.

The primary components of the trial sequence are presented in Figure 1. Each trial began with a number display (two‐digit number or three‐digit number) at the center of the screen for 2000 ms, followed by a central fixation cross and three square placeholders for 500 ms. The center position of each placeholder was at a fixed interval of 4 cm. After the placeholders, a 250‐ms blank screen was rendered on the screen. Subsequently, the target faces in the center placeholder and the distractor faces in the left or right placeholder were presented on the screen for 2000 ms. Within this 2000 ms, participants were asked to try their best to ignore distracting faces and judge the emotional valence of target faces as quickly and accurately as possible. If the target faces expressed anger, participants were asked to press the “X” key. Conversely, if the target faces expressed happiness, responses were made on the keyboard by pressing the “Z” key. To ensure the accuracy of the counting task throughout the experiment, a digital on‐screen keyboard for collecting mouse button responses was presented in the center of the screen. Participants had to use this keyboard to enter the final calculation results. In addition, in order to avoid participant fatigue, “press the space bar to start” would appear in the center of the screen before the start of each trial, and the participants would enter the next trial after pressing the space bar to respond. This setting, which was controlled by the participants themselves, could avoid high error rates (Liu, 2007).

### Data analysis

For data analysis, participants were excluded based on two basic criteria: (a) accuracy level below 50% for the counting task (Petrucci & Pecchinenda, 2016); and (b) accuracy level below 70% for the emotional flanker task. Reaction times (RTs) from trials with errors were excluded, and RTs less than 200 ms or slower than 2.5 *SD*s above the mean for each participant were excluded as outliers. Accuracy and RTs were analyzed using repeated‐measures analysis of variance (ANOVA). The 2 × 3 × 2 × 2 repeated‐measures ANOVA included target face (angry, happy), distractor face (angry, happy, neutral), and WM load (low, high) as the within‐subjects factors and group (HSA, LSA) as the between‐subjects factor. Partial η‐squared values (η_p_^2^) were used to determine the effect sizes for the ANOVA. A significance level of *p* < .05 was used for all analyses.

## RESULTS

The mean accuracy for the emotional flanker task in the HSA and LSA groups is shown in Table 1. The ANOVA conducted on this measure included within‐subjects factors for target face (angry, happy), distractor face (angry, happy, neutral), and WM load (low, high) and between‐subjects factors for group (HSA, LSA). The main effect of group was significant, *F*(1, 68) = 6.54, *p* = .01, η_p_^2^ = .09, indicating that the accuracy of the HSA group was significantly lower than that of the LSA group. The results showed a significant main effect of WM load, *F*(1, 68) = 57.02, *p* < .001, η_p_^2^ = .46, with accuracy on the emotional flanker task significantly lower under high WM load than under low WM load. There was a significant main effect of distractor face, *F*(2, 136) = 15.75, *p* < .001, η_p_^2^ = .19, and a significant main effect of target face, *F*(1, 68) = 17.60, *p* < .001, η_p_^2^ = .21. The results showed a significant two‐way interaction between group and distractor face, *F*(2, 136) = 4.21, *p* = .02, η_p_^2^ = .06, between WM load and distractor face, *F*(2, 136) = 6.73, *p* < .01, η_p_^2^ = .09, and between target face and distractor face, *F*(2, 136) = 3.36, *p* = .03, η_p_^2^ = .05. In addition, we observed a significant three‐way interaction among WM load, distractor face, and group, *F*(2, 136) = 5.7, *p* < .01, η_p_^2^ = .08, and among WM load, distractor face, and target face, *F*(2, 136) = 3.30, *p* = .04, η_p_^2^ = .05. Importantly, there was a significant four‐way interaction among WM load, distractor face, target face, and group, *F*(2, 136) = 6.55, *p* < .01, η_p_^2^ = .09. To further examine this interaction, separate repeated‐measures ANOVAs were conducted for the HSA and LSA conditions.

**TABLE 1:** Mean accuracy for the emotional flanker task in the HSA and LSA groups (M ± *SD*)

WM load	Distractor face	Target face	HSA group (*n* = 34)	LSA group (*n* = 36)
High	Angry	Angry	0.82 ± 0.12	0.86 ± 0.09
		Happy	0.89 ± 0.09	0.88 ± 0.08
	Happy	Angry	0.80 ± 0.11	0.85 ± 0.08
		Happy	0.82 ± 0.12	0.89 ± 0.08
	Neutral	Angry	0.85 ± 0.09	0.87 ± 0.10
		Happy	0.86 ± 0.10	0.89 ± 0.06
Low	Angry	Angry	0.88 ± 0.09	0.93 ± 0.07
		Happy	0.91 ± 0.08	0.95 ± 0.06
	Happy	Angry	0.84 ± 0.11	0.91 ± 0.08
		Happy	0.92 ± 0.09	0.93 ± 0.06
	Neutral	Angry	0.86 ± 0.11	0.92 ± 0.08
		Happy	0.89 ± 0.09	0.94 ± 0.06

Abbreviations: HSA, high social anxiety; LSA, low social anxiety; WM, working memory.

In the HSA group, repeated‐measures ANOVA showed that the main effect of WM load was significant, *F*(1,33) = 19.85, *p* < .001, η_p_^2^ = .38. The main effect of the distractor face was significant, *F*(2, 66) = 15.49, *p* < .001, η_p_^2^ = .32. Bonferroni‐corrected pairwise comparisons revealed that the accuracy produced by happy distractors was significantly lower than that produced by neutral and angry distractors (*p*s < .05), whereas the accuracy produced by angry and neutral distractors did not differ (*p* = .08). There was a significant main effect of target face, *F*(1, 33) = 10.67, *p* < .01, η_p_^2^ = .24. Specifically, post‐hoc comparisons showed that accuracy was lower for the angry targets than that for the happy targets (*p* < .01). The results showed a significant two‐way interaction between WM load and distractor face, *F*(2, 66) = 7.72, *p* < .01, η_p_^2^ = .19, and between target face and distractor face, *F*(2, 66) = 3.58, *p* = .03, η_p_^2^ = .10. The interaction between WM load and target face was not significant, *F*(1, 33) = 2.76, *p* = .11. In addition, the three‐way interaction among WM load, target face, and distractor face was significant, *F*(2, 66) = 7.08, *p* < .01, η_p_^2^ = .18. Simple effects analyses showed that under high WM load, the HSA group exhibited significantly lower accuracy for angry targets compared to happy targets in the presence of angry distractor faces, *F*(1, 33) = 14.41, *p* < .01, η_p_^2^ = .30, whereas there was no significant difference between happy and angry targets in the other interference conditions (*p*s > .05). This simple effects analysis also revealed that in the low WM load condition, the accuracy of angry targets was significantly lower than that of happy targets in the angry and happy distractors conditions (*p*s < .05); in the neutral distractors condition, there was no significant difference in accuracy between angry and happy targets (*p* = .06).

In the LSA group, repeated‐measures ANOVA showed that the main effect of WM load was significant, *F*(1, 35) = 39.49, *p* < .001, η_p_^2^ = .53. The main effect of target face was significant, *F*(1, 35) = 6.76, *p* = .01, η_p_^2^ = .16, revealing that angry targets showed higher accuracy than happy targets (*p* = .01). However, the main effect of distractor face was not significant, *F*(2, 70) = 2.25, *p* = .11. Furthermore, the interaction between all variables was not significant (*p*s >.05).

Mean reaction times for the emotional flanker task in the HSA and LSA groups are presented in Table 2. A repeated‐measures ANOVA for RTs showed a significant main effect of group, *F*(1, 68) = 5.56, *p* = .02, η_p_^2^ = .08, indicating that the HSA group performing the flanker task had longer RTs than the LSA group. The main effect of WM load was significant, *F*(1, 68) = 65.19, *p* < .001, η_p_^2^ = .49, suggesting that participants responded faster when performing the modified flanker task under low WM load than under high WM load. The main effect of target face was significant, *F*(1, 68) = 15.99, *p* < .001, η_p_^2^ = .19. Performing the emotional flanker task for angry targets resulted in longer reaction times (RTs) than for happy targets. The main effect of distractor face, *F*(2, 136) = 4.59, *p* = .01, η_p_^2^ = .06, was statistically significant. Further pairwise comparisons revealed no significant difference in RTs between angry and happy distractors (*p* > .05), but both were significantly higher than neutral distractors (*p*s < .05). There were significant two‐way interactions between group and target face, *F*(1, 68) = 4.44, *p* = .04, η_p_^2^ = .06, between WM load and target face, *F*(1, 68) = 6.56, *p* = .01, η_p_^2^ = .09, and between target face and distractor face, *F*(2, 136) = 4.92, *p* = .01, η_p_^2^ = .07. The analysis also yielded a significant three‐way interaction between target face, WM load, and group, *F*(1, 68) = 4.65, *p* = .04, η_p_^2^ = .06.

**TABLE 2:** Mean reaction times for the emotional flanker task in HSA and LSA groups (M ± *SD*)

WM load	Distractor face	Target face	HSA group (*n* = 34)	LSA group (*n* = 36)
High	Angry	Angry	983 ± 129	923 ± 98
		Happy	952 ± 140	915 ± 88
	Happy	Angry	988 ± 126	925 ± 83
		Happy	956 ± 144	904 ± 95
	Neutral	Angry	980 ± 126	929 ± 92
		Happy	932 ± 150	893 ± 86
Low	Angry	Angry	946 ± 119	860 ± 97
		Happy	916 ± 131	868 ± 98
	Happy	Angry	950 ± 128	868 ± 96
		Happy	923 ± 125	866 ± 93
	Neutral	Angry	954 ± 128	864 ± 107
		Happy	908 ± 139	859 ± 103

Abbreviations: HSA, high social anxiety; LSA, low social anxiety; WM, working memory.

**FIGURE 2:**
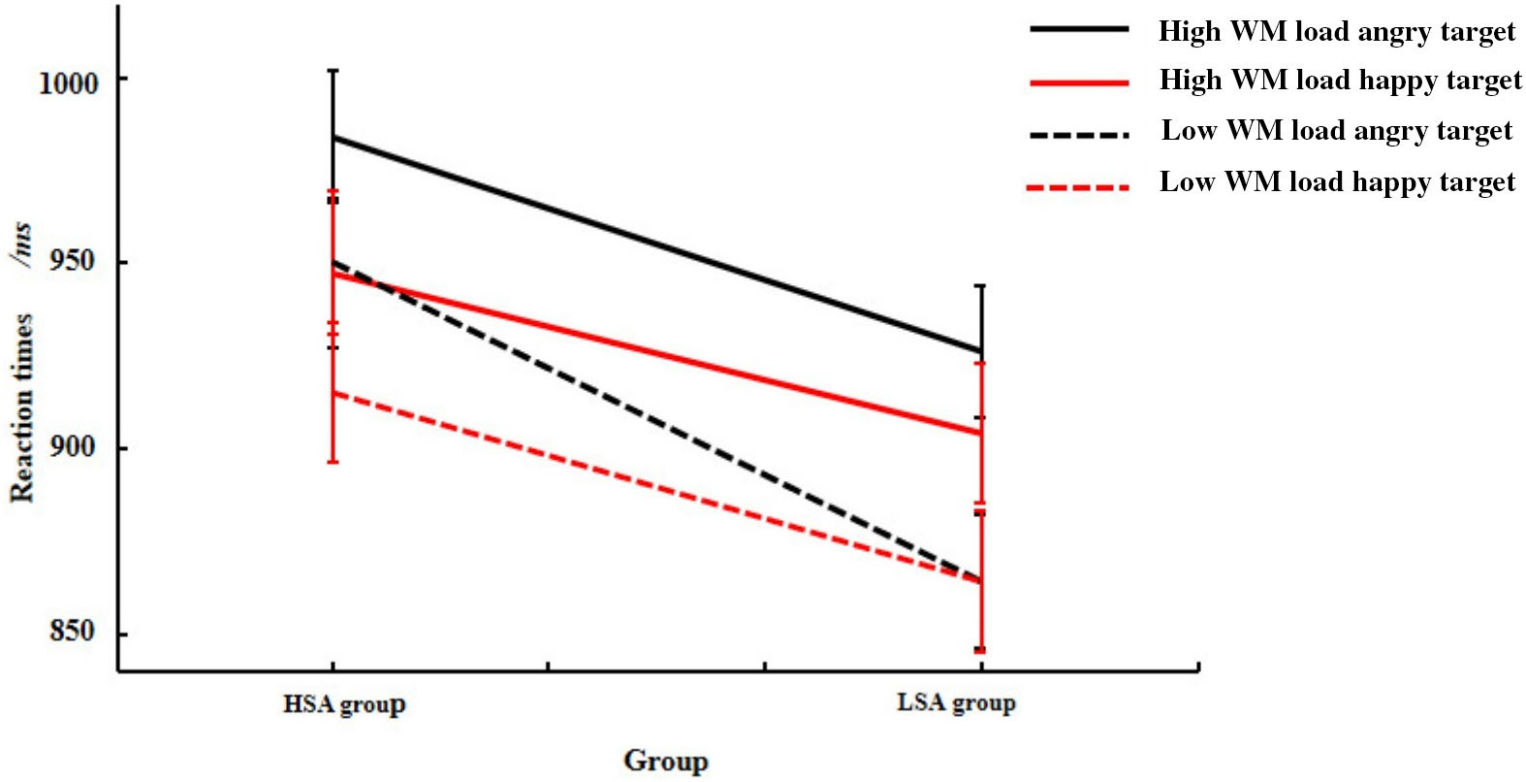
Mean reaction times (RTs) to target faces in the high social anxiety (HSA) and low social anxiety (LSA) groups under high and low working memory (WM) loads. Error bars reflect standard error of the mean (*SEM*). Regardless of WM load, the HSA group had significantly longer RTs to angry targets than to happy targets, whereas this result was observed in the LSA group only under high WM load.

For the three‐way interaction (Figure 2), further simple effects analysis showed that the HSA group had significantly longer RTs to angry targets than to happy targets regardless of the WM load (*ps* < .001). The LSA group had significantly longer RTs to angry targets than to happy targets under high WM load, *F*(1, 68) = 6.48, *p* = .01, η_p_^2^ = .09, whereas there was no significant difference in RTs between angry targets and happy targets under low WM load (*p* = .10).

## DISCUSSION

The present study used a modified flanker task to explore the effect of WM load on selective attention to emotional faces for the HSA group versus the LSA group. This modified flanker task was employed for the first time to investigate the role of cognitive control in the top‐down regulation of selective attention in SA individuals. The results found that the HSA group exhibited lower accuracy and longer RTs on the flanker task compared to the LSA group, which suggests that the poorer performance of the HSA group may be related to impaired cognitive control (Goldin et al., 2009; Morrison & Heimberg, 2013). The results also showed that both groups of participants had lower accuracy and longer RTs under high WM load than under low WM load. This finding suggests that manipulating WM load is effective. Compared to counting forward in multiples of two, the task of subtracting backward in multiples of seven is more challenging and requires more cognitive resources, resulting in poorer performance.

In this study, HSA participants showed interference from both angry and happy distractors under low WM load, resulting in significantly lower accuracy for angry targets compared to happy targets. However, under the high WM load, HSA participants only showed interference from angry distractors and had a lower accuracy for angry targets. In addition, LSA participants did not show interference from any distractors under either WM load, and there was no significant difference in the accuracy between angry and happy targets. The results also showed that the RTs of the HSA group to angry targets were significantly slower than those to happy targets, regardless of WM load. Compared to HSA participants, LSA participants had longer RTs to angry targets than to happy targets, but only under high WM load. These results indicate that the threat‐related attentional bias of HSA individuals is not affected by load manipulation, but is mainly driven by bottom‐up attention.

The current study found that the overall performance of the HSA participants was worse than that of the LSA participants on the emotional flanker task. It is generally accepted that the cognitive control function of SA individuals is impaired (Goldin et al., 2009; Morrison & Heimberg, 2013), resulting in poorer attentional inhibition than healthy individuals (Liang, 2021). The modified flanker task in this study presented the target and distractor at a pre‐specified location and ensured their spatial separation, creating the most favorable conditions for efficient top‐down target selection (Petrucci & Pecchinenda, 2016). However, it was not difficult to speculate from the results that the cognitive control performance of HSA individuals was still not optimistic. Moreover, the attention control theory (ACT) claims that the adverse effect of anxiety on attention control was exacerbated by the increase in WM task demands (Eysenck et al., 2007). Therefore, adding a secondary task to the primary task, or increasing the difficulty of the task, may increase the WM load and enhance the adverse effect of anxiety on attention control (Moriya & Tanno, 2010). In this study, requiring participants to complete both the continuous counting task and the emotional flanker task may exacerbate the adverse effect of anxiety on attention control.

The current research results in HSA individuals are consistent with previous findings using the face–word interference task, which also reported interference from emotional distractors (Pecchinenda et al., 2015). The current results show that in terms of accuracy, HSA individuals showed interference from angry distractors in both WM conditions. In the flanker paradigm, participants are only asked to judge the central target stimuli and do not have to reallocate their attention to the flanker stimuli (Tannert & Rothermund, 2020). Despite instructions to ignore flanker stimuli, the results showed that HSA individuals still allocated attention resources to angry distractors. These findings also provide some support for ACT, which suggests that anxiety enhances the bottom‐up attention system and damages the top‐down attention system (Eysenck et al., 2007). Thus, angry faces with threatening information may be more salient and, with an impaired ability to cognitive control, the threat‐related attentional bias of HSA individuals was more dependent on bottom‐up attention processes.

The present study also found that, in terms of accuracy in the flanker task, HSA individuals showed interference from both angry and happy distractors under low WM load. The results are consistent with the previous framework describing the interaction and competition mechanism between cognition and emotion (Pessoa, 2009, 2010). Because the processing of emotional stimuli might compete for cognitive resources with attention control, participants would only show a processing bias for emotional stimuli (including both positive and negative stimuli) when cognitive load was low. Therefore, if HSA individuals had sufficient cognitive resources, they would also show interference from happy distractors, even if the features of happy faces were not as salient for them. However, LSA individuals did not show interference from emotional distractors under either WM load because LSA individuals had better cognitive control.

The results of the present study showed that under both WM loads, HSA individuals took longer to monitor angry targets during the flanker task. The result is consistent with previous findings that HSA individuals had difficulty in disengaging attention after recognizing angry faces (Liang et al., 2017; Moriya & Tanno, 2011). This finding is well explained by the delayed disengagement hypothesis, which suggests that SA individuals are characterized by difficulty in disengaging their attention from social threats (Amir et al., 2003). HSA individuals found a particular association between angry faces and negative evaluations that threatened them, resulting in their inability to shift their attention in time. Another factor contributing to this finding may be the timing of stimuli presentation, with attentional vigilance to threat in the early stages followed by delayed disengagement from threatening stimuli (Cisler & Koster, 2010; Moriya & Tanno, 2011). The present study used a 2000‐ms stimulus presentation duration, and the observed attentional selectivity may reflect relatively late automatic attentional processing in SA. However, why did HSA individuals spend more time monitoring angry targets but have lower accuracy for angry targets? This may be due to the fact that HSA individuals allocate fewer attentional resources to error monitoring, which plays a crucial role in regulating cognitive functions, such as sustained attention and attentional control (Senderecka, 2018; Xiao et al., 2015), resulting in HSA individuals being less accurate with angry targets.

The present findings have important implications for interventions with SA individuals. First, threat‐related attentional bias is automatic in SA individuals, relying more on bottom‐up attentional processes. Given that involuntary attention to threatening stimuli increases anxiety (MacLeod et al., 2002), SA individuals can be trained to develop a positive interpretation bias, which would help to reduce anxiety. Second, the results of the present study suggest that HSA individuals may allocate fewer attentional resources to error monitoring and that the ability to monitor errors plays a key role in the execution of inhibitory attentional control (Senderecka, 2018; Xiao et al., 2015). It has been demonstrated that interactions between the anterior cingulate cortex, which is involved in error detection, and the frontoparietal attentional control network during the performance of an attentional task help improve task performance in subsequent trials (Walsh et al., 2011).

Considering the following limitations, the interpretation of the results of this study should be interpreted with caution. First, although this study used real faces photographed by professionals, many factors in everyday life, such as hairstyle, skin color, and facial features, may affect face recognition. Therefore, future research could consider using more ecologically dynamic faces as stimuli for experiments. Second, the current research participants were university students, and the extent to which these findings would apply to other populations needs to be further tested. Finally, this study only examined the attentional bias of SA individuals towards emotional faces in selective attention under different WM loads from the behavioral level. Future research will combine neuroscience technology and advanced data analysis to further explore the mechanism of anxiety and WM competition.

Taken together, the current study contributes to the growing literature on threat‐related attentional bias in SA by considering the role of WM resources and emotion in moderating selective attention. The results of the current study indicate that HSA individuals have a threat bias that was unaffected by the WM load manipulation, suggesting that threat‐related attentional biases in HSA individuals rely more on a bottom‐up (stimulus‐driven) system. Supporting ACT, these findings indicate impaired inhibitory control in HSA individuals.

## CONFLICT OF INTEREST STATEMENT

The authors declare no financial conflicts of interest or other benefits to disclose.

## ETHICS STATEMENT

The study was approved by the Institutional Review Board of Jiangxi Normal University. All participants provided written, informed consent before participating in our experiment.
